# Association between first 24-h mean body temperature and mortality in patients with diastolic heart failure in intensive care unit: A retrospective cohort study

**DOI:** 10.3389/fmed.2022.1028122

**Published:** 2022-12-20

**Authors:** Hongyu Xu, Yonggang Xie, Xiaoling Sun, Nianhai Feng

**Affiliations:** ^1^Department of Anesthesiology, Central Hospital of Zibo, Zibo, Shandong, China; ^2^Department of Anesthesiology, The Affiliated Yantai Yuhuangding Hospital of Qingdao University, Yantai, China

**Keywords:** diastolic heart failure, body temperature, hypothermia, mortality, intensive care unit

## Abstract

**Background:**

Body temperature (BT) has been used to evaluate the outcomes of patients with various diseases. In this study, patients with diastolic heart failure (DHF) in the intensive care unit (ICU) were examined for a correlation between BT and mortality.

**Methods:**

This was a retrospective cohort study of the Medical Information Mart for Intensive Care (MIMIC)-IV dataset. A total of 4,153 patients with DHF were included. The primary outcomes were 28-day ICU and higher in-hospital mortality rates. BT was used in the analyses both as a continuous variable and as a categorical variable. According to the distribution of BT, the patients were categorized into three groups (hypothermia BT <36.5°C, normal 36.5°C ≤ BT <37.5°C, and hyperthermia BT ≥37.5°C). Multivariate logistic regression analysis was performed to explore the association between BT and patient outcomes.

**Results:**

The proportions of the groups were 23.6, 69.2, and 7.2%, respectively. As a continuous variable, every 1°C increase in BT was associated with a 21% decrease in 28-day ICU mortality (OR: 0.79, 95% CI: 0.66–0.96, and *p* = 0.019) and a 23% decrease in in-hospital mortality (OR: 0.77, 95% CI: 0.66–0.91; and *p* = 0.002). When BT was used as a categorical variable, hypothermia was significantly associated with both 28-day ICU mortality (OR: 1.3, 95% CI: 1.03–1.65; and *p* = 0.026) and in-hospital mortality (OR: 1.31, 95% CI: 1.07–1.59; and *p* = 0.008). No statistical differences were observed between 28-day ICU mortality and in-hospital mortality with hyperthermia after adjustment.

**Conclusion:**

The first 24-h mean BT after ICU admission was associated with 28-day ICU and in-hospital mortality in patients with DHF. Hypothermia significantly increased mortality, whereas hyperthermia did not.

## Introduction

Body temperature (BT) is a routinely measured vital sign that has been used to evaluate outcomes in patients with various diseases, such as sepsis (1), trauma (2), and multiorgan failure (3). BT abnormalities, including hypothermia and hyperthermia, are the most common symptoms in critically ill patients and the rationale for patient assessment and management (4, 5). Several previous reports claim that, under various circumstances, hypothermia can be used to predict adverse outcomes independently (6, 7). Similarly, hyperthermia has been demonstrated to increase mortality in critically ill patients (8, 9).

Diastolic heart failure (DHF) is defined as a clinical syndrome of heart failure (HF) with a preserved left ventricular ejection fraction (EF) (0.50 or more) without major valve diseases (10). It can be found in approximately one-third of patients with heart failure seen by clinicians. Patients with DHF are more likely to be women, elderly, and have increased blood pressure (11). Unfortunately, some patients with DHF may be underestimated, which hinders timely treatment because of their seemingly normal EF.

A previous study demonstrated that hypothermia is an independent sign of all-cause mortality and cardiovascular (CV) death in patients admitted with worsening HF and reduced EF (12). However, the relationship between BT and mortality among patients with DHF in the intensive care unit (ICU) remains unclear, and BT at a single point in time inevitably increases the risk of deviation (13). Hence, this study aimed to investigate the association between the mean BT of the first 24-h and 28-day ICU mortality and in-hospital mortality of patients with DHF in the ICU from the data collected in a large public clinical database.

## Methods

### Study cohort and data source

This study is reported according to the Strengthening the Reporting of Observational Studies in Epidemiology (STROBE) statement (14). This retrospective study used data collected from the MIMIC-IV (https://mimic.mit.edu/) database (15). In an update to MIMIC-III (16), the database contains comprehensive information (demographics, laboratory results, nursing progress notes, intravenous medications, fluid balance, and other clinical variables) of patients in a Tertiary Academic Medical Center in Boston, MA, USA between 2008 and 2019. Specific diseases were documented according to The International Classification of Diseases, the ninth version (ICD-9) and the tenth version (ICD-10), by hospital staff upon patient discharge. The database is supposed to help a wide variety of healthcare research, and all researchers who have successfully completed the “protecting human subjects” training are authorized to use it. One author of this study, HX, completed the Collaborative Institutional Training Initiative examination (certification number 40287955) and is entitled to access the database for data extraction. None of the patients with MIMIC-IV were identified, and written authorization was not required.

### Study population

**Figure 1:**
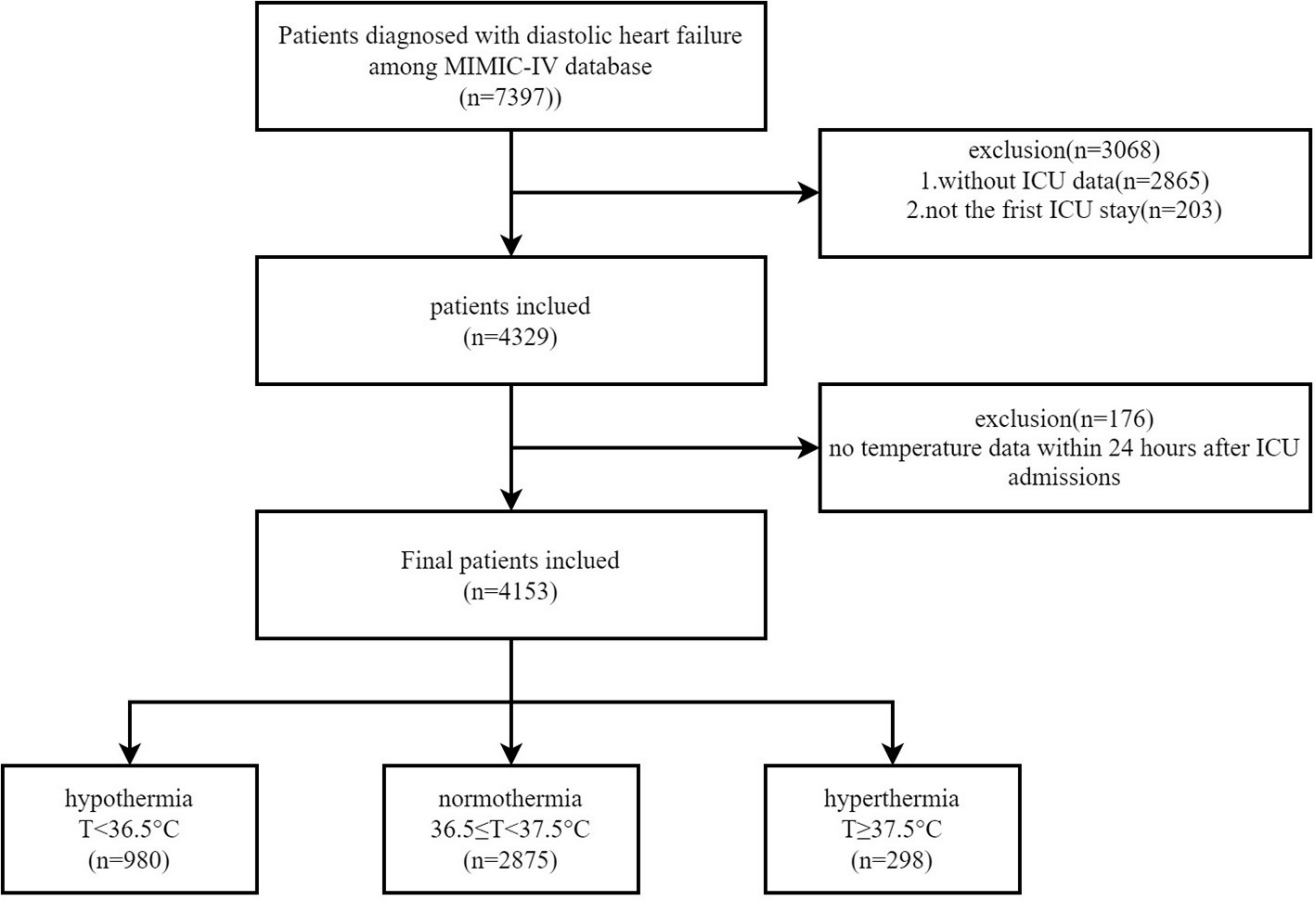
A flowchart of patient selection.

A total of 257,366 individuals were included in the MIMIC-IV database from 2008 to 2019, of whom 50,048 were admitted to the ICU. A total of 7,397 patients with DHF in the MIMIC-IV database were identified using ICD-9 codes 42830, 42831, 42832, and 42833 and ICD-10 codes 1503, 15030, 15031, 15032, and 15033. The study included individuals above the age of 18 years. Because the same patient might have been admitted to the ICU several times, only the first ICU record was analyzed. Patients without ICU data and BT data within 24 h of ICU admission were excluded. Patients who spent <24 h in the ICU were also excluded. Ultimately, only 4,153 patients remained in this study (Figure 1).

### Data extraction

Data extraction was performed using PostgreSQL (version 9.6) and Navicat Premium (version 12.0) software. We obtained the BT of each individual within 24 h of ICU admission by following these steps. First, we searched “temperature” from the table named “d_items,” and then obtained item “223762.” Second, we used this item to obtain the BT from the chart called “chartevents.” Each individual had a different measurement time. After removing the duplicates, we calculated the average BT of each individual using the Excel tool. We divided the patients into three groups based on the temperature performance and the existing research (17–20): hypothermia (BT <36.5°C), normal (36.5°C ≤ BT <37.5°C), and hyperthermia (BT ≥37.5°C). We obtained the survival information for the last outcome variable from the table named “patients.” From the table named “admissions,” we obtained the data regarding the length of hospital and ICU stays (21).

Taking demographics, comorbidities, scoring systems, laboratory tests, and vital signs as variables, we observed the first 24-h ICU records of patients to evaluate the average of vital signs and laboratory tests. Potential bias was prevented by the exclusiveness of data analysis in cases where variables with missing values exceeded 30%.

The following information was gathered: sex, age, ethnicity, marital status, weight, platelets, white blood cells (WBC), albumin, blood urea nitrogen (BUN), international normalized ratio (INR), partial thromboplastin time (PTT), anion gap, bicarbonate, calcium, chloride, sodium, potassium, glucose, heart rate (HR), systolic blood pressure (SBP), respiratory rate (RR), saturation of peripheral oxygen (SPO_2_), sequential organ failure assessment (SOFA) score, acute physiology score (APS) III, Charlson comorbidity index (CCI), urine output (UO) on the 1st day, and fluid input (FI) on the 1st day. The CCI is a scoring system used to quantify comorbidities (including myocardial infarction, congestive heart failure, peripheral vascular disease, cerebrovascular disease, dementia, chronic pulmonary disease, rheumatic disease, peptic ulcer disease, liver disease, diabetes, paraplegia, renal disease, malignant cancer, metastatic solid tumor, and acquired immunodeficiency syndrome).

### Statistical analysis

Continuous variables were divided into normally and non-normally distributed variables. Normally distributed continuous variables are presented as mean ± standard deviation, and non-normally distributed variables are described as median ± interquartile range (IQR). Continuous variables were compared among the groups using a Student's *t*-test or one-way analysis of variance (ANOVA). Categorical variables were described as frequencies or percentages, and the chi-square test was used for comparisons. The Kruskal–Wallis test was applied to assess the significance of differences among different BT groups (the clinical cutoff point). Univariate and multivariable logistic regression analyses were employed to study the relationship between the first 24-h mean BT and 28-day ICU mortality and in-hospital mortality. Variables were chosen based on a relationship in the univariate analysis (*p* < 0.05), clinical judgment, or a change in effect estimate of more than 10%. In the multivariate models, we adjusted only for sex, age, marital status, and ethnicity in Model 1. In Model 2, we adjusted for Model 1 by adding laboratory data such as weight, WBC, albumin, BUN, creatinine, INR, PTT, anion gap, and bicarbonate, calcium, chloride, and sodium levels. In Model 3, we further adjusted for HR, SBP, RR, SpO2, and CCI.

Multivariate regression models were used to conduct trend tests with the median value of each BT group as a continuous variable. Tests were performed to check the results and observe the possibility of non-linearity. To address the non-linearity of the first 24-h mean BT with 28-day ICU and in-hospital mortalities, a smooth curve fitting was conducted. Subgroup analyses were performed using stratified logistic regression. Continuous variables were first changed to categorical variables in accordance with the clinical cutoff points, and then, an interaction test was performed. The adjustments and interactions of the subgroups were examined using the likelihood ratio tests. The subgroups included sex (men or women), age (<75 or ≥75 years), marital status (married, single, divorced, or widowed), ethnicity (Black, White, or other), HR (<80 or ≥80 bpm), weight (<80 or ≥80 kg), the presence of diabetes (yes or on), the presence of MI (yes or on), and the presence of renal disease (yes or no).

Based on five replications and a chained equation approach method of the MI procedure in R, multiple imputations were used to explain the missing data of <30%. The details of the missing values are shown in Supplementary Table S1.

The statistical software package R 3.3.2 (http://www.R-project.org, The R Foundation) and Free Statistics version 1.6.1 were used for all the analyses. A two-tailed test was conducted, and a *p*-value of <0.05 was considered significantly important.

## Results

### Baseline characteristics of patients

This study included 4,153 patients with DHF. The baseline characteristics of these selected patients are presented in Table 1 based on the clinical cutoff point of BT. In total, 980 patients (23.6%) had hypothermia, 298 (7.2%) had hyperthermia, and 2,875 (69.2%) were normal. Overall, the age of the patients was 76.1 ± 12.8 years on average and included approximately 1,857 (44.7%) men. Patients with hypothermia were older and had lower weight, higher CCI scores, higher BUN levels, lower white blood cell count, slower heart rate and respiratory rate, and higher 28-day ICU and in-hospital mortalities. In addition, the SOFA and ApsIII scores were higher with a higher BT. Meanwhile, patients with hyperthermia had longer lengths of stay in the ICU and hospital.

**Table 1:** Baseline characteristics of the patients with diastolic heart failure.

**Variables**	**Over all**	**BT <36.5°C**	**36.5 ≤ BT <37.5°C**	**BT ≥37.5°C**	***p*-value**
	**(*n* = 4,153)**	**(*n* = 980)**	**(*n* = 2,875)**	**(*n*=298)**	
**Gender (%)**					0.105
Men	1,857 (44.7)	417 (42.6)	1,293 (45)	147 (49.3)	
Women	2,296 (55.3)	563 (57.4)	1,582 (55)	151 (50.7)	
Age (years)	76.1 ± 12.8	78.2 ± 12.2	75.8 ± 12.8	71.8 ± 13.3	<0.001
**Ethnicity (%)**					0.005
Black	585 (14.1)	133 (13.6)	404 (14.1)	48 (16.1)	
White	2,964 (71.4)	734 (74.9)	2,036 (70.8)	194 (65.1)	
Other	604 (14.5)	113 (11.5)	435 (15.1)	56 (18.8)	
**Marital status (%)**					<0.001
Married	1,875 (45.1)	409 (41.7)	1,315 (45.7)	151 (50.7)	
Single	899 (21.6)	199 (20.3)	621 (21.6)	79 (26.5)	
Divorced	360 (8.7)	74 (7.6)	256 (8.9)	30 (10.1)	
Widowed	1,019 (24.5)	298 (30.4)	683 (23.8)	38 (12.8)	
Weight (kg)	83.2 ± 27.1	79.3 ± 26.2	83.9 ± 26.7	89.5 ± 31.8	<0.001
UO first 24 h (L)	2.1 (1.1, 3.7)	2.0 (0.9, 3.7)	2.1 (1.1, 3.8)	1.8 (1.0, 3.4)	0.019
FI first 24 h (L)	3.9 (2.2, 6.7)	3.9 (2.1, 6.6)	3.9 (2.2, 6.8)	3.9 (2.3, 6.4)	0.681
**Laboratory tests**					
PLT (10∧9/L)	201.5 (150.5, 264.5)	204.2 (147.5, 271.1)	201.5 (152.2, 263.0)	196.2 (146.6, 253.8)	0.422
WBC (10∧9/L)	10.1 (7.5, 13.7)	9.7 (7.0, 13.5)	10.1 (7.5, 13.6)	11.3 (8.1, 16.3)	<0.001
Albumin (g/dl)	3.0 ± 0.7	3.0 ± 0.7	3.1 ± 0.7	2.9 ± 0.7	<0.001
BNU (mmol/L)	28.5 (19.0, 45.5)	32.0 (20.5, 51.0)	27.5 (18.5, 44.0)	27.2 (17.0, 43.2)	<0.001
INR	1.3 (1.1, 1.7)	1.3 (1.1, 1.9)	1.3 (1.1, 1.6)	1.3 (1.1, 1.7)	<0.001
PTT (s)	32.5 (28.1, 41.4)	33.2 (28.3, 43.2)	32.3 (28.1, 40.7)	31.6 (27.6, 40.1)	0.013
Anion gap (mmol/L)	13.7 ± 4.0	13.9 ± 4.8	13.6 ± 3.8	13.6 ± 3.9	0.279
Bicarbonate (mmol/L)	26.1 ± 5.6	25.8 ± 6.1	26.2 ± 5.4	25.9 ± 5.6	0.113
Calcium (mmol/L)	8.4 ± 0.9	8.4 ± 0.9	8.5 ± 0.9	8.4 ± 0.9	0.009
Chloride (mmol/L)	102.5 ± 6.4	102.8 ± 6.7	102.3 ± 6.2	103.7 ± 6.8	<0.001
Sodium (mmol/L)	138.4 ± 4.9	138.3 ± 4.9	138.3 ± 4.8	139.4 ± 4.8	0.002
Potassium (mmol/L)	4.2 ± 0.7	4.2 ± 0.7	4.2 ± 0.7	4.1 ± 0.7	0.019
Glucose (mmol/L)	7.4 (6.2, 9.3)	7.3 (6.2, 8.9)	7.4 (6.2, 9.3)	7.6 (6.4, 9.6)	0.043
**Vital sign**					
HR (bpm)	83.1 ± 15.7	80.4 ± 15.6	83.1 ± 15.4	91.5 ± 16.3	<0.001
SBP (mmHg)	120.0 ± 18.0	117.0 ± 18.2	121.1 ± 17.9	119.7 ± 16.8	<0.001
RR (bmp)	19.8 ± 3.8	19.0 ± 3.6	19.8 ± 3.7	21.6 ± 4.4	<0.001
SpO2(%)	96.5 ± 2.5	96.5 ± 3.0	96.5 ± 2.3	96.4 ± 2.4	0.91
**Score system**					
SOFA	5.0 (3.0, 7.0)	5.0 (3.0, 7.0)	4.0 (2.0, 6.0)	6.0 (4.0, 9.0)	<0.001
Aps III	47.0 (37.0, 61.0)	52.0 (40.0, 69.0)	46.0 (36.0, 58.0)	54.0 (40.2, 72.8)	<0.001
CCI	6.0 ± 2.2	6.3 ± 2.2	5.9 ± 2.2	5.8 ± 2.3	<0.001
**Comorbidity**					
Myocardial infarct, *n* (%)	285 (6.9)	77 (7.9)	188 (6.5)	20 (6.7)	0.368
Cerebrovascular disease, *n* (%)	262 (6.3)	56 (5.7)	190 (6.6)	16 (5.4)	0.48
Peripheral vascular disease, *n* (%)	321 (7.7)	87 (8.9)	212 (7.4)	22 (7.4)	0.306
Chronic pulmonary disease, *n* (%)	1,185 (28.5)	288 (29.4)	833 (29)	64 (21.5)	0.019
Diabetes, *n* (%)	518 (12.5)	117 (11.9)	347 (12.1)	54 (18.1)	0.009
Renal disease, *n* (%)	1,264 (30.4)	341 (34.8)	833 (29)	90 (30.2)	0.003
Severe liver disease, *n* (%)	52 (1.3)	18 (1.8)	34 (1.2)	0 (0)	0.02
Malignant cancer, *n* (%)	304 (7.3)	70 (7.1)	206 (7.2)	28 (9.4)	0.361
**Outcome**					
LOS hospital (day)	3.4 ± 4.6	3.4 ± 4.9	3.2 ± 4.3	4.8 ± 5.7	<0.001
LOS ICU (day)	9.3 ± 7.9	9.0 ± 7.9	9.3 ± 7.9	10.9 ± 7.4	0.002
28-day ICU mortality, *n* (%)	517 (12.4)	173 (17.7)	302 (10.5)	42 (14.1)	<0.001
In-hospital mortality, *n* (%)	795 (19.1)	247 (25.2)	487 (16.9)	61 (20.5)	<0.001

Data are denoted as mean ± SD or median (IQR) for skewed variables or numbers (proportions) for categorical variables. BT, body temperature; UO, urine output; FI, fluid intake; PLT, Platelets; WBC, white blood cells; BNU, blood urea nitrogen; INR, international normalized ratio; PTT, partial thromboplastin time; HR, heart rate; SBP, systolic blood pressure; RR, respiratory rate; SpO2, saturation of peripheral oxygen; SOFA, sequential organ failure assessment score; Aps III, acute physiology score III; CCI, Charlson comorbidity index; LOS, length of stay; ICU, intensive care unit.

### Univariate and multivariate analyses

In the unadjusted analysis, the first 24-h mean BT as a continuous variable was significantly related to 28-day ICU mortality (OR 0.6, 95% CI 0.51–0.71, and *p* < 0.001) and in-hospital mortality (OR: 0.66, 95% CI: 0.57–0.76, and *p* < 0.001) (Table 2). Age, length of stay in the ICU and hospital, weight, WBC, INP, PTT, anion gap, renal disease, severe liver disease, malignant cancer, the SOFA score, CCI, HR, SBP, RR, and albumin, BUN, creatinine, bicarbonate, calcium, potassium, SpO2 levels were all associated with 28-day ICU mortality and in-hospital mortality. We categorized BT into three groups to calculate, with the normal group as reference: the ORs for 28-day ICU mortality of the hypothermia and hyperthermia groups were 1.83 (95% CI: 1.49–2.24, and *p* < 0.001) and 1.4 (95% CI: 0.99–1.98, and *p* = 0.059), respectively. The ORs for in-hospital mortality of the hypothermia and hyperthermia groups were 1.65 (95% CI: 1.39–1.97, and *p* < 0.001) and 1.26 (95% CI: 0.94–1.7, and *p* = 0.126), respectively.

**Table 2:** Results of univariate analysis of 28-day ICU mortality and in-hospital mortality.

**Variable**	**28-day ICU mortality**		**in-hospital mortality**	
	**OR (95%CI)**	***P*** **-value**	**OR (95%CI)**	***P*** **-value**
BT (°C)	0.6 (0.51–0.71)	<0.001	0.66 (0.57–0.76)	<0.001
36.5 ≤ BT <37.5°C	ref		ref	
BT <36.5°C	1.83 (1.49–2.24)	<0.001	1.65 (1.39–1.97)	<0.001
BT ≥37.5°C	1.4 (0.99–1.98)	0.059	1.26 (0.94–1.7)	0.126
Age (years)	1.03 (1.02–1.04)	<0.001	1.02 (1.01–1.03)	<0.001
LOS hospital	0.98 (0.97–0.99)	0.007	1 (0.99–1.01)	0.795
LOS ICU	1.04 (1.03–1.06)	<0.001	1.05 (1.03–1.06)	<0.001
Weight (kg)	0.99 (0.98–0.99)	<0.001	0.99 (0.99–0.99)	<0.001
WBC (10∧9/L)	1.04 (1.02–1.05)	<0.001	1.02 (1.01–1.03)	<0.001
Albumin (g/dl)	0.54 (0.47–0.62)	<0.001	0.43 (0.38–0.49)	<0.001
BNU (mmol/L)	1.02 (1.01–1.02)	<0.001	1.01 (1.01–1.02)	<0.001
Creatinine (mmol/L)	1.07 (1.03–1.12)	0.002	1.12 (1.08–1.16)	<0.001
INR	1.23 (1.16–1.3)	<0.001	1.19 (1.13–1.25)	<0.001
PTT (s)	1.01 (1.01–1.02)	<0.001	1.01 (1.01–1.01)	<0.001
Anion gap (mmol/L)	1.07 (1.05–1.09)	<0.001	1.07 (1.05–1.09)	<0.001
Bicarbonate (mmol/L)	0.95 (0.94–0.97)	<0.001	0.95 (0.94–0.97)	<0.001
Calcium (mmol/L)	0.86 (0.77–0.95)	0.003	0.77 (0.71–0.85)	<0.001
Potassium (mmol/L)	1.21 (1.07–1.36)	0.003	1.15 (1.04–1.28)	0.008
HR, bpm	1.02 (1.02–1.03)	<0.001	1.02 (1.01–1.02)	<0.001
SBP (mmHg)	0.96 (0.95–0.97)	<0.001	0.98 (0.97–0.98)	<0.001
RR, bpm	1.09 (1.07–1.12)	<0.001	1.07 (1.05–1.09)	<0.001
Renal disease, *n* (%)	1.4 (1.15–1.7)	0.001	1.46 (1.25–1.72)	<0.001
Severe liver disease, *n* (%)	2.38 (1.26–4.49)	0.007	1.9 (1.05–3.43)	0.035
Malignant cancer, *n* (%)	2.14 (1.6–2.85)	<0.001	1.81 (1.39–2.35)	<0.001
Cerebrovascular disease, *n* (%)	1.42 (1.01–1.99)	0.046	1.25 (0.92–1.68)	0.152
Peripheral vascular disease, *n* (%)	1.47 (1.08–2)	0.014	1.15 (0.87–1.52)	0.334
SpO2 (%)	0.87 (0.84–0.9)	<0.001	0.92 (0.89–0.95)	<0.001
SOFA	1.33 (1.29–1.36)	<0.001	1.23 (1.2–1.26)	<0.001
CCI	1.18 (1.14–1.23)	<0.001	1.11 (1.07–1.15)	<0.001

Data presented are ORs and 95% CIs. BT, body temperature; LOS, length of stay; WBC, white blood cells; BNU, blood urea nitrogen; INR, international normalized ratio; PTT, partial thromboplastin time; HR, heart rate; SBP, systolic blood pressure; RR, respiratory rate; SpO2, saturation of peripheral oxygen; SOFA, sequential organ failure assessment score; APS III, acute physiology score III; CCI, Charlson comorbidity index.

Multivariable logistic analysis was used to explore the adjusted association among the first 24-h mean BT, 28-day ICU mortality, and in-hospital mortality. BT as a continuous variable was correlated with 28-day in-ICU mortality and in-hospital mortality in all three models after adjusting for confounders. In the full variables adjusted model, every 1°C increase in BT was associated with a 21% decrease in 28-day ICU mortality and a 23% decrease in in-hospital mortality; the adjusted ORs were 0.79 (95% CI: 0.66–0.96, and *p* = 0.019) and 0.77 (95% CI: 0.66–0.91, and *p* = 0.002), respectively. When analyzed as a categorical variable, the hypothermia group was both relevant to an increased risk of 28-day ICU mortality and in-hospital mortality after adjustment in Model 3; the reference was the normal group, and the adjusted ORs for 28-day ICU mortality and in-hospital mortality were 1.3 (95% CI: 1.03–1.65, and *p* = 0.026) and 1.31 (95% CI: 1.07–1.59, and *p* = 0.008), respectively. Moreover, the adjusted ORs were significant in all the models (Table 3). However, there was no sign of independent correlation in the hyperthermia group with 28-day ICU mortality and in-hospital mortality after multivariable adjustment; the ORs for 28-day ICU mortality and in-hospital mortality in Model 3 were 1.3 (95% CI: 0.88–1.93, and *p* = 0.187) and 1.13 (95% CI: 0.81–1.57, and *p* = 0.463), respectively.

**Table 3:** Multivariable logistic model analysis of BT with 28-day ICU mortality and in-hospital mortality.

**Outcomes**	**n.event_%**	**Univariate**		**Model 1**		**Model 2**		**Model 3**	
		**Crude OR (95% CI)**	***p*-value**	**Adjusted OR (95% CI)**	***p*-value**	**Adjusted OR (95% CI)**	***p*-value**	**Adjusted OR (95% CI)**	***p*-value**
**28-day ICU mortality**									
BT (°C)	517 (12.4)	0.6 (0.51–0.71)	<0.001	0.65 (0.54–0.77)	<0.001	0.78 (0.65–0.94)	0.009	0.79 (0.66–0.96)	0.019
36.5 ≤ BT <37.5°C	302 (10.5)	Ref		Ref		Ref		Ref	
BT <36.5°C	173 (17.7)	1.83 (1.49–2.24)	<0.001	1.72 (1.4–2.11)	<0.001	1.39 (1.11–1.73)	0.004	1.3 (1.03–1.65)	0.026
BT ≥37.5°C	42 (14.1)	1.4 (0.99–1.98)	0.059	1.58 (1.11–2.24)	0.011	1.48 (1.02–2.14)	0.039	1.3 (0.88–1.93)	0.187
*p* for trend			<0.001		0.001		0.161		0.233
**In-hospital mortality**									
BT (°C)	795 (19.1)	0.66 (0.57–0.76)	<0.001	0.69 (0.59–0.8)	<0.001	0.8 (0.68–0.93)	0.005	0.77 (0.66–0.91)	0.002
36.5 ≤ BT <37.5°C	487 (16.9)	Ref		Ref		Ref		Ref	
BT <36.5°C	247 (25.2)	1.65 (1.39–1.97)	<0.001	1.59 (1.34–1.9)	<0.001	1.33 (1.1–1.6)	0.003	1.31 (1.07–1.59)	0.008
BT ≥37.5°C	61 (20.5)	1.26 (0.94–1.7)	0.126	1.36 (1–1.83)	0.047	1.26 (0.92–1.74)	0.149	1.13 (0.81–1.57)	0.463
*p* for trend			<0.001		0.001		0.083		0.057

Data presented are ORs and 95% CIs. BT, body temperature. Model 1 adjusted for gender, age, marital status, and ethnicity; model 2 adjusted for model 1 + weight, WBC, albumin, BUN, creatinine, INR, PTT, anion gap, bicarbonate, calcium, chloride, and sodium; model 3 adjusted for model 2+HR, SBP, RR, SpO2, renal disease, severe liver disease, malignant cancer, myocardial infarction, cerebrovascular disease, peripheral vascular disease, diabetes, chronic pulmonary disease, and CCI.

### Results of non-linearity of BT and mortality

**Figure 2:**
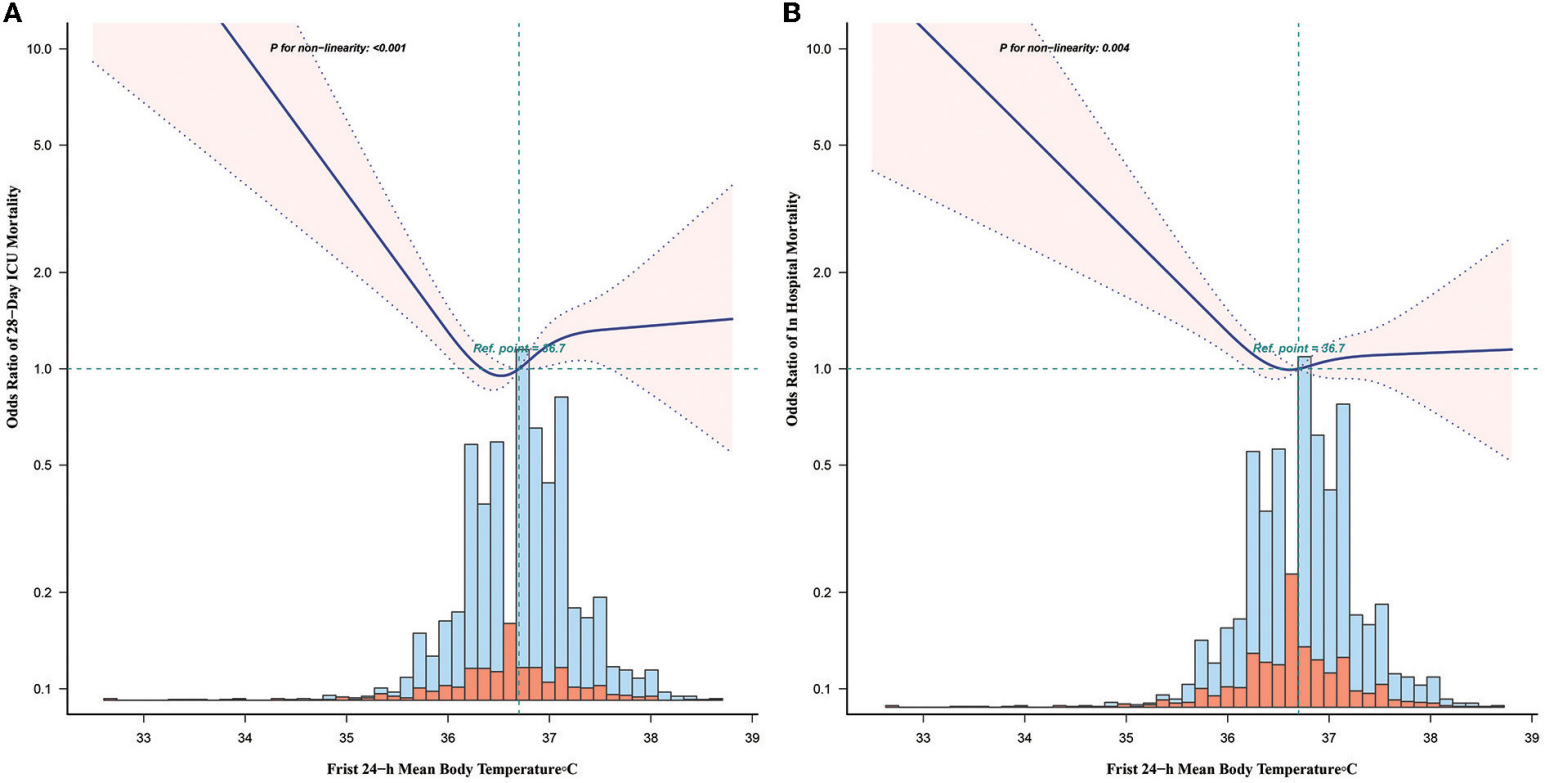
Non-linear relationship between BT and 28-day ICU mortality **(A)** and in-hospital mortality **(B)**. Adjustment factors included gender, age, marital status, ethnicity, weight, WBC, albumin, BUN, INR, PTT, anion gap, bicarbonate, calcium, chloride, sodium, HR, SBP, DBP, MBP, RR, SpO2, the presence of renal disease, the presence of severe liver disease, the presence of malignant cancer, the presence of myocardial infarction, the presence of cerebrovascular disease, the presence of peripheral vascular disease, the presence of diabetes, the presence of chronic pulmonary disease, and CCI.

In the current study, we described the relationship between BT and 28-day ICU mortality and in-hospital mortality in a non-linear model (Figure 2). The smooth curve and the results of the logistic proportional hazards regression model with cubic spline functions demonstrated a non-linear relationship between BT and mortality after adjusting for sex, age, marital status, ethnicity, weight, WBC, albumin, BUN, creatinine, INR, PTT, anion gap, bicarbonate, calcium, chloride, sodium, HR, SBP, RR, SpO_2_ levels, and CCI. The *P*-value for non-linearity is <0.001.

### Subgroup analyses

**Figure 3:**
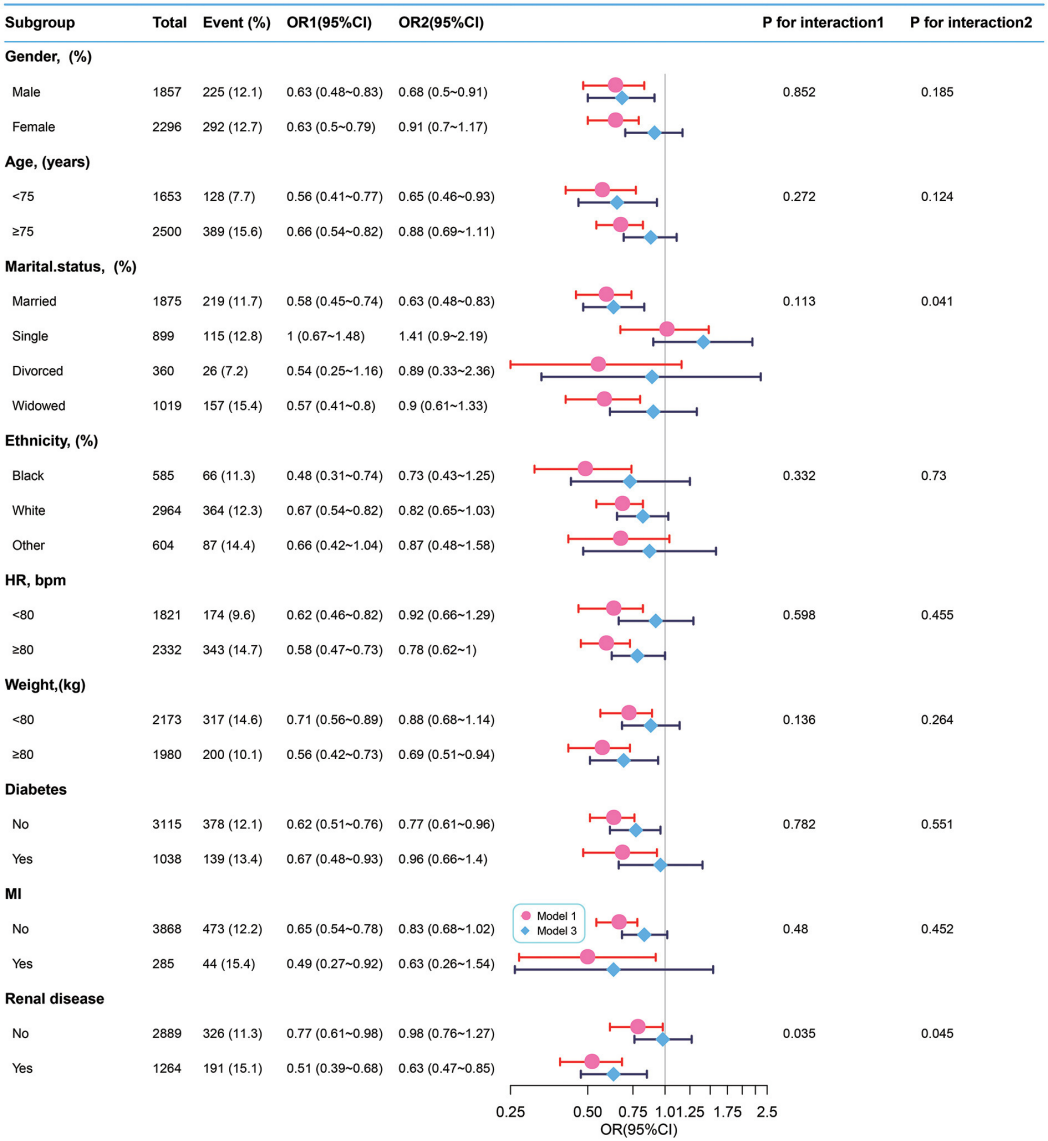
Subgroup analyses of BT and 28-day ICU mortality and in-hospital mortality. Adjustment factors included gender, age, marital status, ethnicity, weight, WBC, albumin, BUN, INR, PTT, anion gap, bicarbonate, calcium, chloride, sodium, HR, SBP, DBP, MBP, RR, SpO2, the presence of renal disease, the presence of severe liver disease, the presence of malignant cancer, the presence of myocardial infarction, the presence of cerebrovascular disease, the presence of peripheral vascular disease, the presence of diabetes, the presence of chronic pulmonary disease, and CCI. BT, body temperature; HR, heart rate; MI, myocardial infarct.

**Figure 4:**
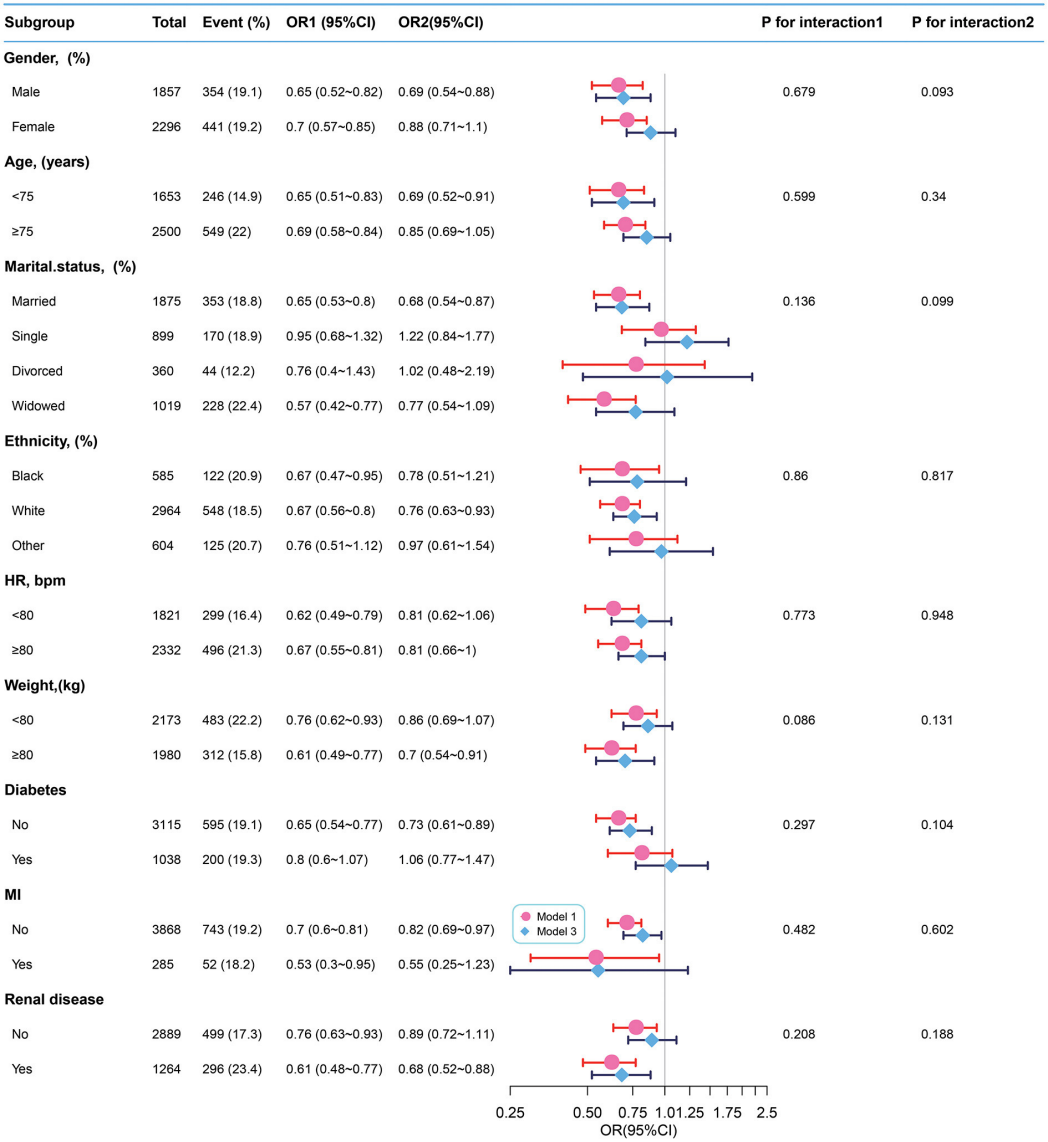
Subgroup analyses of BT and in-hospital mortality. Adjustment factors included gender, age, marital status, ethnicity, weight, WBC, albumin, BUN, INR, PTT, anion gap, bicarbonate, calcium, chloride, sodium, HR, SBP, DBP, MBP, RR, SpO2, the presence of renal disease, the presence of severe liver disease, the presence of malignant cancer, the presence of myocardial infarction, the presence of cerebrovascular disease, the presence of peripheral vascular disease, the presence of diabetes, the presence of chronic pulmonary disease, and CCI. BT, body temperature; HR, heart rate; MI, myocardial infarct.

We used sex, age, ethnicity, marital status, HR, weight, and the presence of diabetes, MI, and renal disease as stratification variables to observe the trend of effect sizes between BT with 28-day ICU mortality and in-hospital mortality in Models 1 and 3 (Figures 3, 4). Consistent results were observed in both Models 1 and 3 (Supplementary Tables S2–S5). We noted that only a few variables showed an interaction between BT and 28-day ICU mortality, including marital status (*P*-values for interaction = 0.041) and absence of renal disease (*P*-values for interaction = 0.045). However, no stratification variables showed an interaction between BT and in-hospital mortality in the two models.

## Discussion

In this retrospective study, we demonstrated the association between the first 24-h mean BT after ICU admission and 28-day ICU mortality and in-hospital mortality among patients with DHF in the ICU. Hypothermia (BT <36.5°C) had significantly increased mortality; however, no statistical associations were observed between hyperthermia (BT ≥37.5°C) and mortality after adjusting the variables.

As a vital sign value, BT can be used to independently assess prognostic risk (5). A previous study found that hypothermia was seemingly more harmful than hyperthermia because it increased 1-year mortality (22) and hypothermia was associated with a significantly higher mortality in patients with worsening HF (12), sepsis (23), frailty in older adults in the ED (7) and after CABG (24). These results are consistent with our results when BT was analyzed as a categorical variable. The reason might be that hypothermia produces significant physiological changes (such as a left shift of the oxygenation curve, a decline in coagulation function, and arrhythmia), which could aggravate tissue hypoxia and multiple organ functions (25).

However, the conclusions of some analyses were inconsistent with our findings. Wade et al. revealed that hypothermia and hyperthermia during hospital admission are associated with increased in-hospital mortality (26). Furthermore, a retrospective study reported that, in patients with infection, an elevated BT peak in the first 24-h ICU admission was associated with a reduction in in-hospital mortality (27). Hyperthermia was thought to be an adaptive physiological response, whereas hypothermia was thought to be a maladaptive response (28). Romanovsky et al. (29) suggested that fever in the mild-to-moderate stage of the disease was a measure to clean the pathogen. In the late stage, the disease has already developed, so hypothermia might be the outcome of a more severe condition with multiorgan failure, inflammatory processes, and hormonal factors involving thermoregulation, or low body temperature might directly result in adverse consequences (12, 30, 31).

Heart failure (HF) is a common but complex syndrome caused by a wide variety of etiologies, initially called systolic heart failure (SHF) and DHF. More recently, based on the calculated ejection fraction (EF) of 50%, HF was described as HFrEF and HFpEF (32). One report showed that patients with DHF might have a similar EF to those with HFpEF (33). In March 2021, the *European Journal of Heart Failure* published the first version of the Universal Definition and Classification of Heart Failure, led by the Heart Failure Society of America (HFSA) (34), ending the history of inconsistent definitions and classifications of heart failure. The data for our study were extracted from the MIMIC-IV, in which patient information was recorded from 2008 to 2019. The database uses a previous standard to diagnose DHF. Patients with DHF were documented according to The International Classification of Diseases ninth version (ICD-9) codes 42830, 42831, 42832, and 42833 and tenth version (ICD-10) codes 1503, 15030, 15031, 15032, and 15033. In a large clinical trial, Payvar et al. revealed the relationship between low BT and adverse consequences in hospitalized patients with worsening HF. In an average of 9.9 months following the study of 4,108 patients after discharge, lower body temperature at randomization showed a linear correlation with an increased possibility of CV death or HF rehospitalization (12). Casscells and colleagues identified that hypothermia at hospital arrival was significantly related to higher in-hospital mortality, observed in another retrospective study of 291 HF hospitalized patients for HF exacerbation (35). The patients in these previous studies were all patients with HFrEF, and few studies have explored HFpEF. The patients in our study were diagnosed with DHF, whose EF was ≥0.50 and similar to HFpEF.

Compared with previous studies, our study has several advantages. First, the focus of the study was on the influence of overall body temperature changes in the patients on the first 24 h in the ICU instead of the adverse outcomes associated with a single time point. Therefore, our study might be particularly valuable for the temperature management of patients in the ICU. Second, this was an observational study and, therefore, vulnerable to potential confounding factors. A statistical adjustment was performed to reduce the residual confounders as much as possible. Third, the target independent variables were classified as both continuous and categorical variables. Such clarification could decrease contingency in data analysis and increase the robustness of the results. Fourth, the subgroup analysis suggested that the consistent relationships between BT and 28-day ICU mortality and in-hospital mortality were significant in the different subgroups in this study.

To the best of our knowledge, this is the first study to observe an independent relationship between the first 24-h mean BT and 28-day ICU mortality and in-hospital mortality in patients with DHF in the ICU. This would help highlight the significance of abnormal BT and might help reduce the adverse consequences of hypothermia in these patients. Although BT can be obtained easily, lower LOS values are often neglected by clinical staff, and this study would inspire further concern regarding this simple and easily available finding.

However, the present study has some limitations. First, the sample size of the hyperthermia group was relatively small, which needs to be enlarged, and a multicenter database is necessary for verification in the future. Second, the BT varied according to the measurement site. Clarification of the method and site in the MIMIC-IV database is not clear, which might lead to deviations in the results. Further research is needed to clarify these details. Third, as a retrospective study, this study used cases of DHF diagnosed according to administrative diagnosis codes. Although this study used the first sequence of diagnoses, it is still possible that there are false associations caused by misclassifications. Fourth, in the MIMIC-IV database, there was no mention of varying degrees of diastolic dysfunction, and this study may not have compared the differences for varying degrees of diastolic dysfunction. Finally, the study was conducted at a single institution and might have selection bias, and further studies are necessary to investigate the external generalizability.

## Conclusion

This retrospective observational study proved that the mean BT of patients in the first 24 h in ICU was related to 28-day ICU mortality and in-hospital mortality in patients with DHF. Hypothermia significantly increased mortality, whereas hyperthermia did not. The significance of BT monitoring is worthy of attention for improving risk stratification and the treatment selection process for patients with DHF undergoing clinical treatment.

## Data availability statement

The raw data supporting the conclusions of this article will be made available by the authors, without undue reservation.

## Author contributions

HX conceived the idea, conducted data analysis, and drafted the manuscript. YX interpreted the results and helped to revise the manuscript. XS conducted the data collection. NF conducted data analysis and reviewed the manuscript. All authors read and approved the final manuscript.
