# Supplementary material for: Association between first 24-h mean body temperature and mortality in patients with diastolic heart failure in intensive care unit: A retrospective cohort study
Source: Front Med (Lausanne). 2022 Dec 20;9:1028122. doi: 10.3389/fmed.2022.1028122 (PMC9807784; doi:10.3389/fmed.2022.1028122)
Supplement: Supplementary file 1 [file Table_1.DOCX]

**Table S1** | Details of missing values.


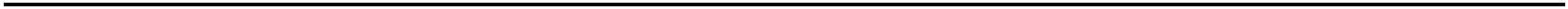

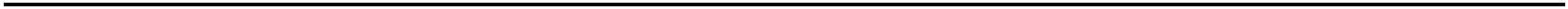

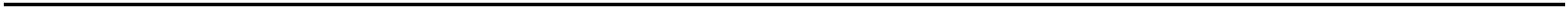


Variable The number of missing values The percent of missing values (%)
Albumin,(g/dl) 386 9.29
Aniongap,(mmol/L) 204 4.91
Bicarbonate,(mmol/L) 198 4.77
BNU, (mmol/L) 16 0.39
Calcium,(mmol/L) 850 20.47
Chloride,(mmol/L) 172 4.14
FI first 24h,(L) 14 0.34
UO first 24h,(L) 121 2.91
Glucose,(mmol/L) 66 1.59
INR 400 9.63
Marital status, (%) 94 2.26
PLT, (10^9/L) 26 0.63
Potassium,(mmol/L) 169 4.07
PTT,(s) 423 10.19
SBP,(mmHg) 12 0.29
Sodium,(mmol/L) 170 4.09
Spo2(%) 2 0.05
WBC, (10^9/L) 27 0.65
Weight,(kg) 45 1.08
BNU, blood urea nitrogen ;UO, Urine output; FI, fluid intake; INR, international normalized ratio; PLT, Platelets;
PTT, partial thromboplastin time; SBP, systolic blood pressure; SpO2, saturation of peripheral oxygen; WBC, white
blood cells.

**Table S2** Subgroup analyses of BT and 28-day ICU mortality in model 1.


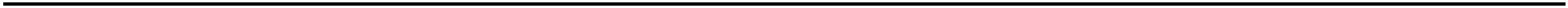

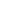

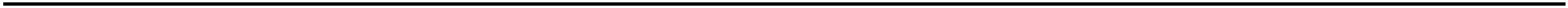


Subgroup Total Event(%) Crude OR (95% CI) Crude P value Adj OR(95%CI) Adj P value P for interaction
Gender, (%)

Male 1857 225 (12.1) 0.63 (0.49~0.82) <0.001 0.63 (0.48~0.83) 0.001 0.852
 Female 2296 292 (12.7) 0.58 (0.46~0.73) <0.001 0.63 (0.5~0.79) <0.001
Age, (years)

<75 1653 128 (7.7) 0.55 (0.4~0.75) <0.001 0.56 (0.41~0.77) <0.001 0.272
 ≥75 2500 389 (15.6) 0.68 (0.55~0.84) <0.001 0.66 (0.54~0.82) <0.001
Marital.status, (%)

Married 1875 219 (11.7) 0.56 (0.44~0.71) <0.001 0.58 (0.45~0.74) <0.001 0.113
 Single 899 115 (12.8) 0.93 (0.63~1.37) 0.71 1 (0.67~1.48) 0.981
 Divorced 360 26 (7.2) 0.49 (0.23~1.03) 0.06 0.54 (0.25~1.16) 0.113
 Widowed 1019 157 (15.4) 0.57 (0.41~0.8) 0.001 0.57 (0.41~0.8) 0.001
Ethnicity, (%)

Black 585 66 (11.3) 0.47 (0.3~0.71) <0.001 0.48 (0.31~0.74) 0.001 0.332
 White 2964 364 (12.3) 0.63 (0.51~0.77) <0.001 0.67 (0.54~0.82) <0.001
 Other 604 87 (14.4) 0.61 (0.39~0.94) 0.024 0.66 (0.42~1.04) 0.076
HR, bpm

<80 1821 174 (9.6) 0.61 (0.46~0.82) 0.001 0.62 (0.46~0.82) 0.001 0.598
 ≥80 2332 343 (14.7) 0.54 (0.43~0.66) <0.001 0.58 (0.47~0.73) <0.001
Weight,(kg)

<80 2173 317 (14.6) 0.69 (0.55~0.86) 0.001 0.71 (0.56~0.89) 0.003 0.136
 ≥80 1980 200 (10.1) 0.54 (0.42~0.7) <0.001 0.56 (0.42~0.73) <0.001
Diabetes

No 3115 378 (12.1) 0.59 (0.49~0.72) <0.001 0.62 (0.51~0.76) <0.001 0.782

Yes 1038 139 (13.4) 0.62 (0.45~0.87) 0.005 0.67 (0.48~0.93) 0.018
MI

No 3868 473 (12.2) 0.61 (0.51~0.73) <0.001 0.65 (0.54~0.78) <0.001 0.48
 Yes 285 44 (15.4) 0.52 (0.29~0.93) 0.027 0.49 (0.27~0.92) 0.027
Renal disease

No 2889 326 (11.3) 0.72 (0.57~0.9) 0.004 0.77 (0.61~0.98) 0.03 0.035
 Yes 1264 191 (15.1) 0.5 (0.38~0.65) <0.001 0.51 (0.39~0.68) <0.001
Adjusted for gender,age,marital status,ethnicity.

**Table S3** Subgroup analyses of BT and 28-day ICU mortality in model 3.


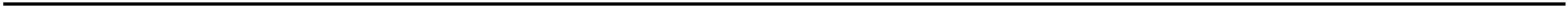

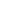

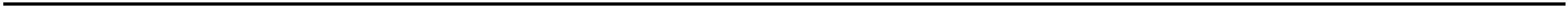


Subgroup Total Event(%) Crude OR (95% CI Crude P value Adj OR(95%CI) Adj P value P for interaction
Gender, (%)

Male 1857 225 (12.1) 0.63 (0.49~0.82) <0.001 0.68 (0.5~0.91) 0.009 0.185
 Female 2296 292 (12.7) 0.58 (0.46~0.73) <0.001 0.91 (0.7~1.17) 0.451
Age, (years)

<75 1653 128 (7.7) 0.55 (0.4~0.75) <0.001 0.65 (0.46~0.93) 0.019 0.124
 ≥75 2500 389 (15.6) 0.68 (0.55~0.84) <0.001 0.88 (0.69~1.11) 0.266
Marital.status, (%)

Married 1875 219 (11.7) 0.56 (0.44~0.71) <0.001 0.63 (0.48~0.83) 0.001 0.041
 Single 899 115 (12.8) 0.93 (0.63~1.37) 0.71 1.41 (0.9~2.19) 0.132
 Divorced 360 26 (7.2) 0.49 (0.23~1.03) 0.06 0.89 (0.33~2.36) 0.808
 Widowed 1019 157 (15.4) 0.57 (0.41~0.8) 0.001 0.9 (0.61~1.33) 0.591
Ethnicity, (%)

Black 585 66 (11.3) 0.47 (0.3~0.71) <0.001 0.73 (0.43~1.25) 0.255 0.73
 White 2964 364 (12.3) 0.63 (0.51~0.77) <0.001 0.82 (0.65~1.03) 0.084
 Other 604 87 (14.4) 0.61 (0.39~0.94) 0.024 0.87 (0.48~1.58) 0.647
HR, bpm

<80 1821 174 (9.6) 0.61 (0.46~0.82) 0.001 0.92 (0.66~1.29) 0.64 0.455
 ≥80 2332 343 (14.7) 0.54 (0.43~0.66) <0.001 0.78 (0.62~1) 0.049
Weight,(kg)

<80 2173 317 (14.6) 0.69 (0.55~0.86) 0.001 0.88 (0.68~1.14) 0.33 0.264
 ≥80 1980 200 (10.1) 0.54 (0.42~0.7) <0.001 0.69 (0.51~0.94) 0.017
Diabetes

No 3115 378 (12.1) 0.59 (0.49~0.72) <0.001 0.77 (0.61~0.96) 0.021 0.551

Yes 1038 139 (13.4) 0.62 (0.45~0.87) 0.005 0.96 (0.66~1.4) 0.819
MI

No 3868 473 (12.2) 0.61 (0.51~0.73) <0.001 0.83 (0.68~1.02) 0.078 0.452
 Yes 285 44 (15.4) 0.52 (0.29~0.93) 0.027 0.63 (0.26~1.54) 0.31
Renal disease

No 2889 326 (11.3) 0.72 (0.57~0.9) 0.004 0.98 (0.76~1.27) 0.883 0.045
 Yes 1264 191 (15.1) 0.5 (0.38~0.65) <0.001 0.63 (0.47~0.85) 0.003
Adjusted for gender,age,marital status,ethnicity,weight,WBC,albumin,BUN,creatinine,INR,PTT,aniongap,bicarbonate,calcium,sodium,
HR,SBP,RR,Spo2,CCI.

**Table S4** Subgroup analyses of BT and in-hospital mortality in model 1.


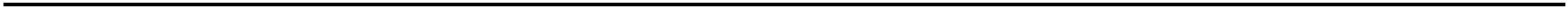

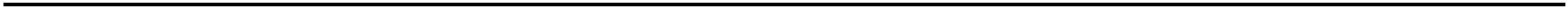

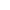

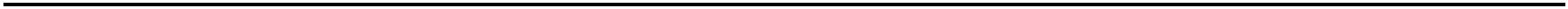


Subgroup Total Event(%) Crude OR (95% C Crude P value Adj OR(95%CI) Adj P value P for interaction
Gender, (%)

Male 1857 354 (19.1) 0.65 (0.52~0.8) <0.001 0.65 (0.52~0.82) <0.001 0.679
 Female 2296 441 (19.2) 0.66 (0.55~0.81) <0.001 0.7 (0.57~0.85) <0.001
Age, (years)

<75 1653 246 (14.9) 0.64 (0.5~0.82) <0.001 0.65 (0.51~0.83) 0.001 0.599
 ≥75 2500 549 (22) 0.7 (0.59~0.85) <0.001 0.69 (0.58~0.84) <0.001
Ethnicity, (%)

Black 1875 353 (18.8) 0.63 (0.51~0.78) <0.001 0.65 (0.53~0.8) <0.001 0.136
 White 899 170 (18.9) 0.93 (0.67~1.29) 0.646 0.95 (0.68~1.32) 0.753
 Other 360 44 (12.2) 0.71 (0.39~1.32) 0.28 0.76 (0.4~1.43) 0.391
Marital.status, (%) 1019 228 (22.4) 0.56 (0.41~0.76) <0.001 0.57 (0.42~0.77) <0.001
 Married

Single 585 122 (20.9) 0.64 (0.45~0.91) 0.012 0.67 (0.47~0.95) 0.025 0.86
 Divorced 2964 548 (18.5) 0.64 (0.54~0.77) <0.001 0.67 (0.56~0.8) <0.001
 Widowed 604 125 (20.7) 0.71 (0.49~1.04) 0.076 0.76 (0.51~1.12) 0.16
HR, bpm

<80 1821 299 (16.4) 0.62 (0.49~0.78) <0.001 0.62 (0.49~0.79) <0.001 0.773
 ≥80 2332 496 (21.3) 0.63 (0.52~0.76) <0.001 0.67 (0.55~0.81) <0.001
Weight,(kg)

<80 2173 483 (22.2) 0.76 (0.62~0.92) 0.006 0.76 (0.62~0.93) 0.007 0.086
 ≥80 1980 312 (15.8) 0.59 (0.47~0.73) <0.001 0.61 (0.49~0.77) <0.001
Diabetes

No 3115 595 (19.1) 0.62 (0.53~0.74) <0.001 0.65 (0.54~0.77) <0.001 0.297

Yes 1038 200 (19.3) 0.75 (0.56~1) 0.049 0.8 (0.6~1.07) 0.137
MI

No 3868 743 (19.2) 0.66 (0.57~0.77) <0.001 0.7 (0.6~0.81) <0.001 0.482
 Yes 285 52 (18.2) 0.56 (0.32~0.97) 0.039 0.53 (0.3~0.95) 0.032
Renal disease

No 2889 499 (17.3) 0.72 (0.59~0.87) 0.001 0.76 (0.63~0.93) 0.007 0.208
 Yes 1264 296 (23.4) 0.6 (0.47~0.75) <0.001 0.61 (0.48~0.77) <0.001
Adjusted for gender,age,marital status,ethnicity.

**Table S5** Subgroup analyses of BT and in-hospital mortality in model 3.


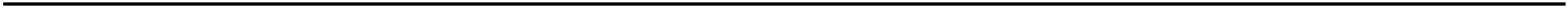

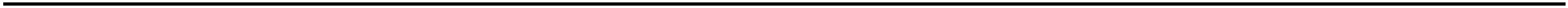

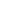

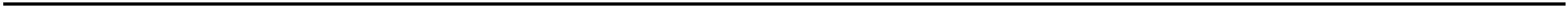


Subgroup Total Event(%) Crude OR (95% C Crude P value Adj OR(95%CI) Adj P value P for interaction
Gender, (%)

Male 1857 354 (19.1) 0.65 (0.52~0.8) <0.001 0.69 (0.54~0.88) 0.003 0.093
 Female 2296 441 (19.2) 0.66 (0.55~0.81) <0.001 0.88 (0.71~1.1) 0.276
Age, (years)

<75 1653 246 (14.9) 0.64 (0.5~0.82) <0.001 0.69 (0.52~0.91) 0.008 0.34
 ≥75 2500 549 (22) 0.7 (0.59~0.85) <0.001 0.85 (0.69~1.05) 0.137
Ethnicity, (%)

Black 1875 353 (18.8) 0.63 (0.51~0.78) <0.001 0.68 (0.54~0.87) 0.002 0.099
 White 899 170 (18.9) 0.93 (0.67~1.29) 0.646 1.22 (0.84~1.77) 0.305
 Other 360 44 (12.2) 0.71 (0.39~1.32) 0.28 1.02 (0.48~2.19) 0.956
Marital.status, (%) 1019 228 (22.4) 0.56 (0.41~0.76) <0.001 0.77 (0.54~1.09) 0.138
 Married

Single 585 122 (20.9) 0.64 (0.45~0.91) 0.012 0.78 (0.51~1.21) 0.267 0.817
 Divorced 2964 548 (18.5) 0.64 (0.54~0.77) <0.001 0.76 (0.63~0.93) 0.007
 Widowed 604 125 (20.7) 0.71 (0.49~1.04) 0.076 0.97 (0.61~1.54) 0.907
HR, bpm

<80 1821 299 (16.4) 0.62 (0.49~0.78) <0.001 0.81 (0.62~1.06) 0.122 0.948
 ≥80 2332 496 (21.3) 0.63 (0.52~0.76) <0.001 0.81 (0.66~1) 0.054
Weight,(kg)

<80 2173 483 (22.2) 0.76 (0.62~0.92) 0.006 0.86 (0.69~1.07) 0.183 0.131
 ≥80 1980 312 (15.8) 0.59 (0.47~0.73) <0.001 0.7 (0.54~0.91) 0.007
Diabetes

No 3115 595 (19.1) 0.62 (0.53~0.74) <0.001 0.73 (0.61~0.89) 0.001 0.104

Yes 1038 200 (19.3) 0.75 (0.56~1) 0.049 1.06 (0.77~1.47) 0.725
MI

No 3868 743 (19.2) 0.66 (0.57~0.77) <0.001 0.82 (0.69~0.97) 0.02 0.602
 Yes 285 52 (18.2) 0.56 (0.32~0.97) 0.039 0.55 (0.25~1.23) 0.147
Renal disease

No 2889 499 (17.3) 0.72 (0.59~0.87) 0.001 0.89 (0.72~1.11) 0.305 0.188
 Yes 1264 296 (23.4) 0.6 (0.47~0.75) <0.001 0.68 (0.52~0.88) 0.003
Adjusted for gender,age,marital status,ethnicity,weight,WBC,albumin,BUN,creatinine,INR,PTT,aniongap,bicarbonate,calcium,sodium,
HR,SBP,RR,Spo2,CCI

| **Table S6** \| The corresponding disease terms of the ICD codes. | | |
| --- | --- | --- |
| version | codes | disease terms |
| ICD-9 | 42830 | Diastolic heart failure, unspecified |
|  | 42831 | Acute diastolic heart failure |
|  | 42832 | Chronic diastolic heart failure |
|  | 42833 | Acute on chronic diastolic heart failure |
| ICD-10 | 1503 | Diastolic (congestive) heart failure |
|  | 15030 | Unspecified diastolic (congestive) heart failure |
|  | 15031 | Acute diastolic (congestive) heart failure |
|  | 15032 | Chronic diastolic (congestive) heart failure |
|  | 15033 | Acute on chronic diastolic (congestive) heart failure |
